# Sugar and Polymer Excipients Enhance Uptake and Splice-Switching Activity of Peptide-Dendrimer/Lipid/Oligonucleotide Formulations

**DOI:** 10.3390/pharmaceutics11120666

**Published:** 2019-12-09

**Authors:** Osama Saher, Taavi Lehto, Olof Gissberg, Dhanu Gupta, Oskar Gustafsson, Samir EL Andaloussi, Tamis Darbre, Karin E. Lundin, C. I. Edvard Smith, Rula Zain

**Affiliations:** 1 Department of Laboratory Medicine, Clinical Research Center, Karolinska Institutet, Karolinska University Hospital Huddinge, SE-141 86 Huddinge, Sweden; taavi.lehto@ki.se (T.L.); olof.gissberg@ki.se (O.G.); dhanu.gupta@ki.se (D.G.); oskar.gustafsson@ki.se (O.G.); samir.el-andaloussi@ki.se (S.E.A.); rula.zain@ki.se (R.Z.); karin.lundin@ki.se (K.E.L.); 2 Department of Pharmaceutics and Industrial Pharmacy, Faculty of Pharmacy, Cairo University, 11562 Cairo, Egypt; 3 Institute of Technology, University of Tartu, 50411 Tartu, Estonia; 4 Department of Chemistry and Biochemistry, University of Bern, Freiestrasse 3, 3012 Bern, Switzerland; tamis.darbre@dcb.unibe.ch; 5 Centre for Rare Diseases, Department of Clinical Genetics, Karolinska University Hospital, SE-171 76 Stockholm, Sweden

**Keywords:** splice-switching oligonucleotide, gene therapy, transfection enhancers, dendrimers, excipients, synergism, BTK, X-linked agammaglobulinemia

## Abstract

Non-viral transfection vectors are commonly used for oligonucleotide (ON) delivery but face many challenges before reaching the desired compartments inside cells. With the support of additional compounds, it might be more feasible for a vector to endure the barriers and achieve efficient delivery. In this report, we screened 18 different excipients and evaluated their effect on the performance of peptide dendrimer/lipid vector to deliver single-stranded, splice-switching ONs under serum conditions. Transfection efficiency was monitored in four different reporter cell lines by measuring splice-switching activity on RNA and protein levels. All reporter cell lines used had a mutated human β-globin intron 2 sequence interrupting the luciferase gene, which led to an aberrant splicing of luciferase pre-mRNA and subsidence of luciferase protein translation. In the HeLa Luc/705 reporter cell line (a cervical cancer cell line), the lead excipients (Polyvinyl derivatives) potentiated the splice-switching activity up to 95-fold, compared to untreated cells with no detected cytotoxicity. Physical characterization revealed that lead excipients decreased the particle size and the zeta potential of the formulations. In vivo biodistribution studies emphasized the influence of formulations as well as the type of excipients on biodistribution profiles of the ON. Subsequently, we suggest that the highlighted impact of tested excipients would potentially assist in formulation development to deliver ON therapeutics in pre-clinical and clinical settings.

## 1. Introduction

Peptide dendrimers were first reported in early 1980s by Denkewalter et al. [1] and Aharoni et al. [2] and while they are nowadays categorized into different classes, full peptide dendrimers are the most interesting, from the delivery point of view. This class is composed of only amino acids, from the core to the outer shell [3]. As oligonucleotide (ON) delivery vectors, peptide dendrimers are usually equipped with lysine, arginine, histidine, or a mixture of these amino acids to protect and deliver genetic material [4,5,6]. Although the replacement of polymeric dendrimers with peptide dendrimers result in reduced cytotoxicity, they still face many biological barriers for effective delivery. For instance, they need to confer adequate serum resistance, cellular uptake, and endosomal escape [7].

To overcome some of the aforementioned barriers, combining different vector structures is a simple, yet efficient strategy. The essence behind such a strategy is the opportunity to achieve synergistic effects on gene delivery while evading possible toxicity- or synthesis-related complications. The vector combination strategy has been successfully applied in a number of studies for the delivery of different cargos [8,9,10,11,12,13].

Regarding single-stranded ON delivery by dendrimers, most studies have focused on using the polyamidoamine dendrimers or other polymeric structures [14,15,16]. However, not much has been reported on investigating the use of peptide dendrimers to deliver single-stranded ONs, particularly, for splice-switching applications. We previously described the synthesis and testing of 20 different peptide dendrimer structures for their ability to deliver splice-switching ONs. The ternary complexes of the Peptide Dendrimer/Lipid/ON were named after the initial of each component i.e., Peptide dendrimer/lipid/ON (PDLO)-complexes [17].

**Figure 1:**
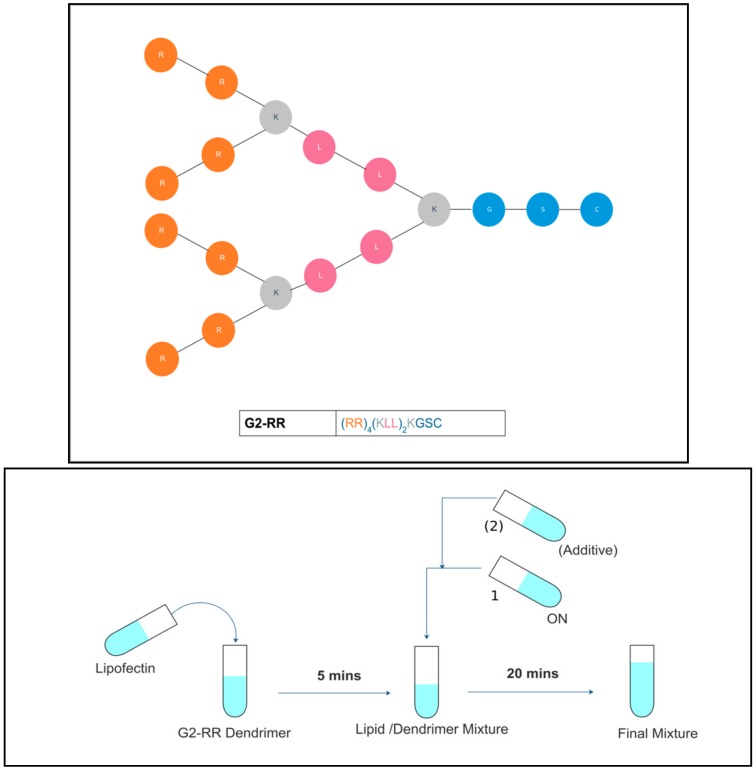
General schematic representation of the G2-RR peptide dendrimer structure and formulation protocol. In the top panel, one-letter code is used to label amino acids incorporated into the dendrimers, where C (Cysteine), S (Serine), and G (Glycine) constitute the core; K (lysine) is the attachment point for building higher generations; and R (Arginine) and L (Leucine) are the building units that constitute the rest of the structure. The nomenclature labels dendrimer by the generation G, whereas the numbers beside the letter indicate in which of the three layers the amino acids motif (LL) is substituted with other amino acid motifs (in here it is RR). In the lower panel, a schematic illustration of the complexation protocols with or without the excipients is shown. Excipient addition (if any) is performed directly after adding the oligonucleotide to the Lipid/dendrimer mixture.

The G2-RR peptide dendrimer (Figure 1) was identified as the lead candidate, since it displayed the highest activity in this study. Yet, the efficacy was compromised in the presence of serum. Although transfection under serum conditions is closer to the in vivo situation, serum has been often reported to negatively affect the transfection efficacy by various mechanisms [18]. Interestingly, the transfection efficiency of the G2-RR PDLO-complex under serum conditions was enhanced upon the addition of sucrose to the formulations [17].

Saccharides and polyol compounds are generally utilized to protect non-viral delivery vectors during lyophilization, as they are hypothesized to form a glassy matrix that immobilizes vectors and prevents aggregations during freezing [19]. However, the particle isolation hypothesis later introduced by Allison et al. rather suggests that lyo-protectants act before freezing the sample [20]. Lower surface tension achieved by these compounds keeps particles dispersed in the unfrozen state; thus, they prevent coalescence and maintain vector efficacy during freezing [20]. Taking into account the particle isolation hypothesis and based on the aforementioned studies, we wanted to investigate the effect of various excipients together with our formulations, in addition to sucrose, which has been previously tested. We assumed that these excipients would increase the possibility of keeping particles dispersed, thereby, potentially maintaining or enhancing the delivery efficacy of the vector.

In this report, we investigated the serum associated transfection efficacy of PDLO-complexes consisting of the reported peptide dendrimer (G2-RR) with Lipofectin (as a lipid component) and the splice-switching ONs to be delivered [17]. To enhance the transfection efficacy under these conditions, we focused on screening 18 different excipients, consisting of sugars and polymers.

The screening involved four different reporter cell lines, in which we monitored transfection efficacy and their effect on cell viability. We also measured the size and zeta potential of these modified formulations. To gain insight into the effects of these excipients, we investigated the behavior of the formulations on the level of cellular uptake, as well as the kinetic profile of the functional effects via monitoring of luciferase activity in the presence or absence of the excipients. Finally, in vivo biodistribution patterns of different formulations were investigated after intravenous injection in mice.

## 2. Materials and Methods

### 2.1. Materials

The 18-mer splice-switching ON with the sequence (5′-CCUCUUACCUCAGUUACA) was synthesized at GE Healthcare. The ON had a fully modified phosphorothioate backbone and 2′-*O*-methyl modified ribose sugar units. 187.15-5LNA (5′-aGacTuaCcaCuuCc) was used for the *BTK* gene cell models [21]. The G2-RR peptide dendrimer (Figure 1) used in the study was synthesized using solid phase synthesis while applying Fmoc/Boc protection strategy, as reported previously [17]. Lipofectin (a liposomal formulation of a 1:1 (*w/w*) ratio of DOTMA (*N*-[1-(2,3-dioleyloxy)- propyl]-*N*,*N*,*N*-trimethylammonium chloride) and DOPE (dioleoyl phosphotidylethanolamine)), and Lipofectamine 2000 (L2000) were both purchased from Invitrogen (Stockholm, Sweden). Polyvinylpyrrolidone “PVP” (average molecular weights: 10, 40, and 55 kDa); Polyvinylalcohol “PVA” (average molecular weights: 18, and 40 kDa); Pluronic F 68 “PF68”; Pluronic F 127 “PF127”; Tween 80 “TW80”; Tween 20 “TW20”; (2-Hydroxypropyl)-β-cyclodextrin “HP-β-CD” and all other sugars were bought from Sigma–Aldrich (Stockholm, Sweden).

### 2.2. Cell Line and Culture Conditions

HeLa Luc/705, HuH7_705, U-2 OS_705, and Neuro 2a_705 reporter cell lines were cultured and maintained in high glucose Dulbecco’s modified Eagle’s medium (DMEM) (Invitrogen) supplemented with 10% fetal bovine serum (FBS) (Invitrogen) at 37 °C, in a humidified incubator with 5% CO_2_. U2OS/EGFPLucBTKint4mut reporter cell line representing additional human disease (X-linked agammaglobulinemia) characterized by mutations in the *BTK* gene [21], was cultured using the same conditions.

### 2.3. ON Transfection of Reporter Cell Lines Under Serum Conditions

One day before transfection, the cells were seeded in sterile, clear bottom, white TC-treated, 96-well plates (Corning^®^, Stockholm, Sweden). Seeding was done at a density of 12 × 10^3^ (HeLa Luc/705), 15 × 10^3^ (HuH7_705), or 11 × 10^3^ (U-2 OS_705, and Neuro 2a_705) to obtain 70–80% confluency/well the next day.

The G2-RR PDLO-complex formulation procedures, N/P ratio, and Lipofectin amounts were the same as described previously [17]. All formulations were prepared in HEPES buffered glucose (HEPES buffered glucose (HBG) buffer/20 mM HEPES, 5% glucose, pH = 7.4). In case of any tested excipient, equal volumes of the concentrated excipient solution were added directly after the ON addition to the mixture, to obtain an isotonic final concentration (Table 1). The formulated mixtures were incubated for 20 min at room temperature, before being added to the cells kept in a full growth medium. A simplified scheme of the preparation process is shown in Figure 1.

Regardless of the formulation type, cell culture media were replaced with 90 µL fresh media before the addition of complexes to cells. The added complexes constituted 10% of the final media volume and were incubated with the cells for 24 h, at 37 °C, in a humidified incubator with 5% CO_2_, before luciferase measurements. Control complexes using Lipofectin or L2000 with the ON were also formulated following manufacturers’ recommendations. Finally, excipient complexes with ON (without dendrimers and lipids) were also tested. For the U2OS/EGFPLucBTKint4mut reporter cell line, seeding was done at a density of 13 × 10^3^ (70–80% confluency/well the next day). The G2-RR PDLO-complex formulation procedures were the same but the middle final ON concentration (25 nM) was used as reported in [21], as was the case for the ON targeting the β-globin site in our previous study [17].

### 2.4. Fluorescence Microscopy

Cy5-labeled ONs of the same chemistry and length were transfected into the HeLa Luc/705 reporter cell line, under serum conditions, as previously mentioned. The G2-RR PDLO-complex, with or without the selected excipients, was used. At the assigned time points (4, 8, and 24 h) the media were aspirated and the cells were rinsed and incubated with DMEM^®^ media-No Phenol Red (Invitrogen). Live cell imaging was performed using a fluorescence microscope (Olympus IX81, Olympus America Inc., Center Valley, PA, USA).

### 2.5. Flow Cytometric Analysis

Cy5-labeled ONs of the same chemistry and length were transfected into the HeLa Luc/705 reporter cell line, under serum conditions, as previously mentioned. G2-RR PDLO-complexes formulated in HBG were tested with or without the selected excipients. At the assigned time points (4, 8, and 24 h), cells in the 96-well plates were trypsinized and re-suspended in DMEM^®^ media supplemented with 10% fetal bovine serum (FBS) (Invitrogen). Cells were then stained with DAPI (0.1 µg/mL), before analysis by flow cytometry on a MACSQuant Analyzer 10 instrument, equipped with three lasers (405, 488, 638 nm; Miltenyi Biotec, Bergisch Gladbach, Germany). DAPI was detected in channel V1 (450/50 nm) and Cy5 in channel R1 (655–730 nm). At least 2000 cells per well were acquired, and the events were gated for single, viable cells. All data were analyzed using the FlowJo software, v10.5.3 (FlowJo LLC, Ashland, OR, USA).

### 2.6. Luciferase Assay

For the lysis and measurement, we followed the same protocol as previously described [17]. DC Protein Assay (Bio-Rad) protocol was applied for the determination of total protein quantity in five microliter lysate. The relative light units (RLU) of luciferase were determined (GloMax^®^ 96 Microplate Luminometer machine-Promega, Stockholm, Sweden) with a 10 s integration time and 2 s delay between injection and measurement. All values were normalized to the total protein quantities measured in micrograms and then further normalized to the values of the untreated cells, to represent the final results as a fold increase in luciferase activity.

### 2.7. RNA Expression Analysis of Splice-Correction

For determination of the percentage corrected luciferase mRNA, total RNA was isolated from the cells using the RNeasy plus kit (QIAGEN, Stockholm, Sweden). Three nanograms of isolated RNA were used in each RT-PCR reaction using a ONE STEP RT-PCR kit (QIAGEN). The total volume was 20 µL and the primers had the following sequences: Fwd-5′-TTGATATGTGGATTTCGAGTCGTC; Rev-5′-TGTCAATCAGAGTGCTTTTGGCG. The program for the RT-PCR was as follows: 55 °C, 35 min, then 95 °C, 15 min, for the reverse transcription step, directly followed by the PCR (94 °C, 30 s, then 55 °C, 30 s, then 72 °C, 30 s) for 30 cycles and finally 72 °C, 10 min, for final extension. The PCR products were analyzed using a 2% agarose gel in 0.5× TBE buffer and visualized by SYBR Gold (Invitrogen, Molecular products) staining. Analysis of gels was done with the Versadoc imaging system with a cooled CCD camera (BioRad, Hercules, CA, USA). Band intensities were analyzed with the Quantity One software (BioRad, Hercules, CA, USA), and the percentage of corrected ON was calculated by normalization against the sum of band intensities of corrected and un-corrected bands (Band intensity of corrected RNA * 100/(Band intensity of corrected RNA + Band intensity of uncorrected RNA).

### 2.8. Effect of Lyophilization on Activity

Lyophilization was conducted for the formulation with or without the selected excipients, for 2 h, using (MAXI-DRY LYO^®^, Allerød, Denmark). The dried products were reconstituted and added to the cells. The lysis of cells and luciferase measurements were performed 24 h after the addition of the reconstituted products.

### 2.9. Particle Size and Zeta Potential Measurements

Freshly prepared complexes were subjected to particle size measurement using Zetasizer Nano ZS machine (Malvern Instruments, Worcestershire, UK). Complexes, with or without excipients, were formulated in HBG and diluted either in HBG or OptiMEM^®^ (Invitrogen, Stockholm, Sweden) The z-average particle size, PDI (polydispersity index), and zeta potential were estimated for each sample. Measurements were performed at least three times per sample in disposable, folded capillary cells (DTS1060).

### 2.10. Cell Viability

The WST-1 assay was used to assess the viability of cells upon treatment with complexes, together with excipients. We followed the same protocol as in [17,26]. Cells were seeded and transfected in 96-well plates. One day after the treatment, the medium was replaced with fresh medium supplemented with WST-1 reagent (dilution 1:10). The cells were incubated for 2 h at 37 °C in a humidified incubator with 5% CO_2_. Absorbance measurements were done at 450 nm with a reference wavelength of 650 nm (SpectraMAX i3x, Molecular Devices, San Jose, CA, USA). Values were expressed as the percentage of the ratio of the absorbance at 450 nm of the treated cells to the untreated cells.

### 2.11. Animal Biodistribution Experiment

G2-RR PDLO-complexes, with or without lead excipients, were freshly prepared using Alexa-568 labeled ON (with the same sequence and chemistry as the ON used in cell experiments). A total of 25 µg Alexa-568-labeled ON were slowly injected intravenously through the tail veins of female NMRI mice either in the form of formulations or ON/HBG buffer mixture (positive control). After 24 h, the animals were sacrificed and the harvested organs were immediately imaged ex-vivo with fluorescence analysis (excitation 570 nm, emission 620 nm) using IVIS Spectrum (Perkin Elmer). Analysis of data and background elimination was done later with the IVIS software (Living Image Software for IVIS). The animal experiments were approved by the Swedish local ethics committee (Approval Number: S4-16; Date: 5 January 2016).

### 2.12. Data Analysis

Data are expressed as mean with a standard error of the mean (SEM). Statistical significance was determined by one or two-way analysis of variance (ANOVA), followed by individual comparisons using Fisher’s least significant difference test (GraphPad Prism 6 Software; GraphPad Software, Inc., San Diego, CA, USA). In all cases, *p* < 0.05 was considered significant.

## 3. Results and Discussion

### 3.1. Sugar Excipients Enhance Transfection Efficiency of G2-RR PDLO-Complexes in HeLa Luc/705 Reporter Cell Line

**Figure 2:**
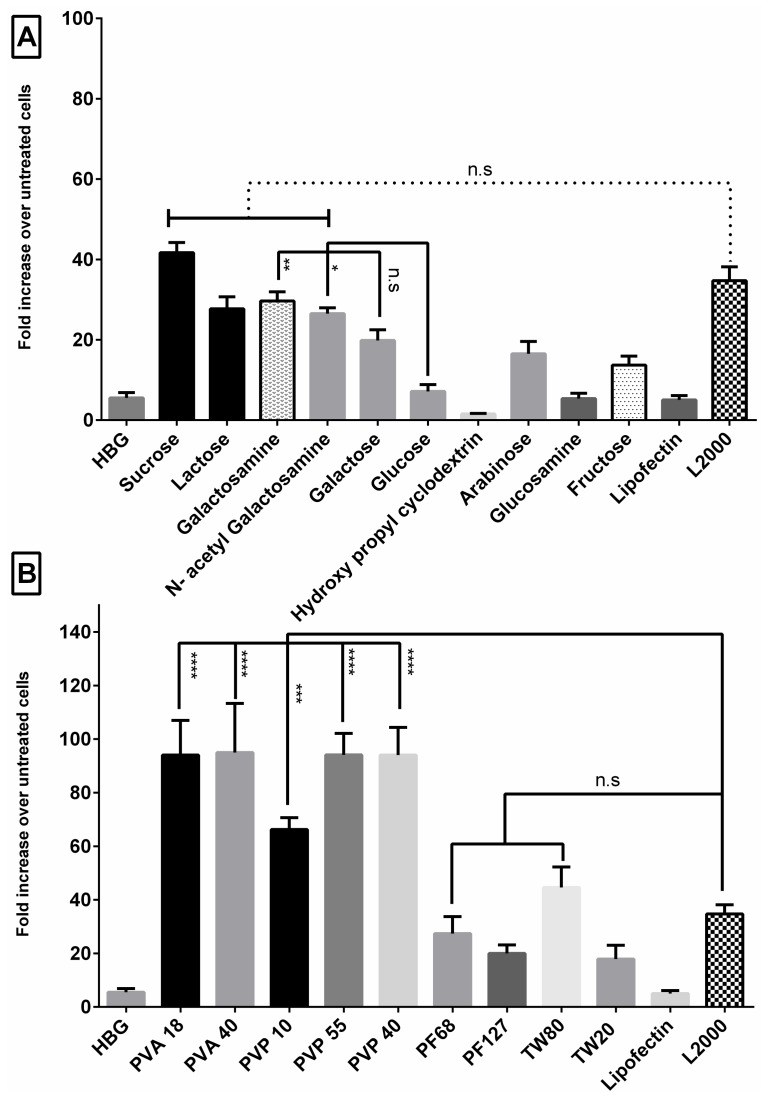
Effect of excipients on transfection efficiency of G2-RR Dendrimer/Lipofectin/Oligonucleotide (PDLO) complexes in HeLa Luc/705 reporter cell line under serum conditions. Fold increase in the luciferase signal normalized by micrograms of total protein of transfected versus untreated cells for the G2-RR PDLO-complexes, (**A**) with or without sugar excipients, and (**B**) with or without polymer excipients. Oligonucleotide (ON) concentration was 0.1 µM and complexes with L2000 or Lipofectin were used as controls. Error bars = SEM (*n* ≥ 3), n.s.: non-significant, * *p* ≤ 0.05, ** *p* ≤ 0.01, *** *p* ≤ 0.001, and **** *p* ≤ 0.0001 (one-way ANOVA, post hoc Fisher’s LSD against Glucose (**A**) or L2000 (**A**,**B**)).

After the development of pLuc/705 splice-switching reporter cell line and demonstrating the possibility to use ON to correct splicing defects [27,28], such a luciferase-based assay has become a commonly used tool to assess vector efficacy to deliver splice-switching ON. We have utilized this assay to investigate the transfection efficiency of G2-RR PDLO-complexes, with or without excipients that were divided into two major classes—sugars and polymers (Table 1). After measuring the luciferase signal and normalizing to the protein content, fold increase in luciferase activity over the untreated cells were calculated (Figure 2). For sugars, we previously reported the use of sucrose excipient that enhanced serum transfection efficacy of PDLO-complexes to a level comparable to Lipofectamine 2000 (L2000) [17]. Therefore, we aimed to extend our investigation to include other sugars in this study. From the panel of various sugars tested, only lactose, galactosamine, and *N*-acetyl galactosamine showed a similar enhancement as sucrose (Figure 2). Splice-correction levels were comparable to L2000 upon transfection with formulations supplemented with lactose, galactosamine, or *N*-acetyl galactosamine with 27-, 29-, 26-fold higher luciferase activity than untreated cells, respectively. Galactose, arabinose, and fructose also had a positive impact on the activity (19-, 16-, 13-fold increase, respectively); however, this increase did not reach the levels comparable to L2000.

Disaccharides are known to be superior in maintaining particle stability and preventing aggregations compared to monosaccharides [29]. Sucrose and lactose are commonly used as protective excipients during lyophilization [30]. Since both compounds showed a comparable enhancement of transfection, we also screened the corresponding monosaccharides. We hypothesized that glucose could be the effective candidate, being the common monosaccharide in both lactose and sucrose. However, glucose did not show any transfection enhancement. Surprisingly, it was the other monosaccharide (fructose or galactose) subunits that had a positive impact on the transfection. In addition, arabinose was tested as an example of a five-carbon lyoprotectant monosaccharide [31], and it showed a comparable activity to fructose and galactose. Finally, the addition of high molecular weight oligosaccharides such as a (2-Hydroxypropyl)-β-cyclodextrin/sucrose mixture to the transfection vector, reduced the transfection efficacy. This was in contrast to previous reports that have highlighted the potential of cyclodextrin or cyclodextrin/sucrose mixtures in increasing cellular uptake and transfection efficiency of ONs [22,32].

From these screenings, it was also evident that the transfection efficiency of galactose was further increased upon replacement with amino sugar analogs, in contrast to glucose (Figure 2A). Transfection efficiency of galactosamine or *N*-acetyl galactosamine was increased by nearly 1.5-fold, compared to unmodified galactose. *N*-acetyl galactosamine (GalNAc) conjugation to siRNA has been previously reported to significantly increase siRNA uptake and achieve a robust gene silencing in hepatocytes, both in cellular and animal models [33,34].

### 3.2. G2-RR PDLO-Complexes Display Transfection Efficiencies Superior to Lipofectamine 2000 (L2000) in the Presence of Polymer Excipients

After characterizing the impact of sugar excipients on the transfection efficiency of G2-RR PDLO-complexes, we next sought to study the potential of various polymeric compounds. Polymer excipients dramatically enhanced the transfection efficiency of the formulations (Figure 2B). It is worth mentioning that the majority of polymer excipients used here were mixed with sucrose to ensure the isotonicity and isoosmolarity of the mixture. Polymer excipients showed almost the same enhancement when used alone (data not shown). PF68 and Tw80 addition to formulations (Figure 2B) produced a comparable activity to L2000 (27- and 44-fold increase over background, respectively). Tw80 surfactants have been reported to exert particle stabilizing effects and they are often used as cryoprotectants [35].

Interestingly, the two highest molecular weights of PVP (40 and 55 kDa) and the two tested molecular weights of PVA (18 and 40 kDa) displayed the best results and were significantly better than L2000. PVA and PVP are widely used in pharmaceutical excipients, and in the context of gene therapy applications they were reported as transfection vectors to deliver plasmids [23]. They have also been used as efficient lyoprotectant excipients for proteins and vector formulations [22,36]. PVP has also been utilized as a lysosomotropic agent [37], and has been used as a stabilizer of monoclonal antibodies [38].

PVP10 showed the lowest activity in its category (66-fold increase over the background), as compared to the higher average molecular weights PVP40 and PVP55 (both showed a 94-fold increase over the background), as depicted in Figure 2B.

Regarding the PVA analogs tested, they all had an impact on the transfection efficiency that seemed to be independent of their molecular weight. Although PVA was previously reported as a transfection vector by itself [23], in our hands different PVA variants had a negligible effect on the transfection efficiency of ONs on their own. In fact, none of the most active polymers or sugars enhanced transfection activity when mixed with the ON without dendrimers/lipid mixture (Supplementary Figure S1). This would rule out the possibility that these excipients influence ON activity by themselves. Similarly, excipients did not enhance the transfection efficiency when added to the dendrimer/ON mix without Lipofectin (Supplementary Figure S2). We previously reported that Lipofectin is an integral component in the formulation mixture and that dendrimers do not have an effect on their own [17]. Interestingly, a great enhancement of the transfection efficacy of the Lipofectin/ON mix was observed upon mixing with PVA excipients [17]. It seemed that the excipients preferentially enhanced vectors that had an initial activity on their own. Additionally, it has been reported that PVA can be adsorbed on the surface of particles, preferentially via the formation of H-bonds [39,40]. Finally, although the transfection efficacy of dendrimers alone was not enhanced by the excipients, PVA had a double enhancement effect in the presence of dendrimers in the formulations (Figure 2B), compared to conditions where PVA was added to the Lipofectin/ON mix only (Supplementary Figure S2).

### 3.3. Splice-Corrected mRNA Levels Confirmed the Enhancing Activity of Excipients on Transfection

**Figure 3:**
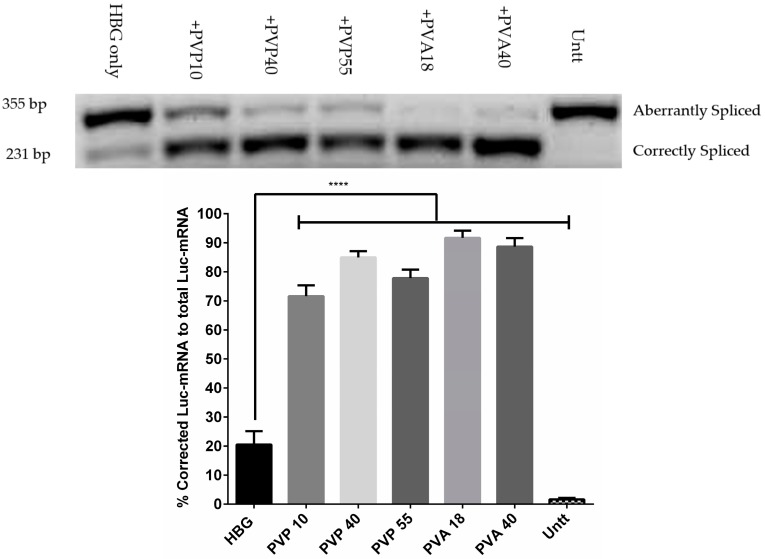
Splice-corrected RNA levels after serum associated transfection of G2-RR Dendrimer/Lipofectin/Oligonucleotide (PDLO) complexes, with or without selected excipients. Upper panel: Representative gel showing the corrected and uncorrected amplicon bands after treatment with PDLO-complexes together with selected excipients. Lower Panel: Levels of corrected RNA after quantification and normalization to total RNA levels. HeLa Luc/705 cells were treated under serum conditions with PDLO-complexes using an oligonucleotide concentration of 0.1 µM for 24 h. Splice-correction levels were calculated by the ratio of corrected band against the sum of the band intensities of the corrected and the un-corrected bands. Error bars = SEM (*n* ≥ 3), **** *p* ≤ 0.0001 (one-way ANOVA, post hoc Fisher’s LSD against HBG result). * HBG = HEPES buffered glucose

Correction levels in RNA are generally examined in parallel to protein correction levels, to determine any inconsistency between the obtained RNA and protein levels. Hence, in addition to investigating the correction of luciferase on the protein level, we wanted to confirm the correction on RNA levels. Figure 3 demonstrates the levels of corrected mRNA after treatment with G2-RR PDLO-complexes together with the selected excipients. The highest correction obtained (91 ± 4.5%) was reached using the PVA18, with the formulations. For all tested polymers, the RNA levels correlated with the protein levels.

### 3.4. PVA Excipients Are More Lyoprotective to the Formulations Compared to the PVP Excipients

Lyophilization is an accepted method to prolong the stability of the formulation solution by converting it into a solid form. However, the lyophilization process itself can negatively affect the activity of the formulations. Since most of the tested excipients are usually used as lyo-protectants, we wanted to study if they could confer protection of the formulations during lyophilization. As shown (Supplementary Figure S3), the transfection efficiency was diminished after lyophilization and reconstitution in the presence of excipients. Luciferase values declined to be around 60-fold over background upon the use of PVA. However, the values dramatically decreased to a much lower level (~30-fold) over the background in the case of PVP excipients, which highlights the superior protective effects of PVA over PVP during the lyophilization/reconstitution process. Additionally, reconstitution with HBG buffer instead of water showed similar results in the case of PVA excipients but lower values with PVP. These results can be explained in the light of a published study indicating that PVA is adsorbed more strongly to the particle surface than PVP due to the higher ability to form H-bonds [39]. This means that PVA would perhaps offer more protection for the particle, and better stabilization.

### 3.5. Excipients Tested Are Non-Toxic to HeLa Luc/705 Cells

**Figure 4:**
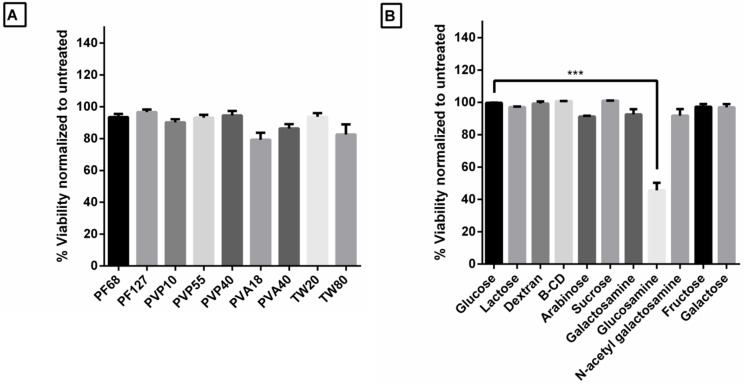
Effect of excipients on cell viability. Viability of HeLa Luc/705 cells after transfection with (**A**) polymer excipients with formulations and (**B**) sugar excipients with formulations. Error bars = SEM (*n* ≥ 3), *** *p* ≤ 0.001 (one-way ANOVA, post hoc Fisher’s LSD against Glucose).

Generally, two important features are required in any delivery vector. It should be effective and should have no or minimal toxicity. In many cases, high activity of a vector is accompanied by toxic effects on the treated cells. Thus, it is important to investigate possible toxicities in any tested vectors. We investigated the formulations, with or without the excipients. In our case, HeLa Luc/705 reporter cell line displayed no sign of toxicity upon transfection of complexes with any polymer excipient, under serum conditions (Figure 4A). Sugar excipients similarly displayed no toxicity, with the exception of glucosamine that caused major toxicity in the tested cells (Figure 4B). It has previously been reported that glucosamine had toxic effects on the tissue cultures of sarcoma cells [41]. Finally, microscopic images of cells treated with formulations containing the best excipients and excipient-free formulations showed no major changes in morphology or confluency (Supplementary Figure S4).

### 3.6. Selected Excipients Enhance Cellular Uptake of the Complexes and Luciferase Correction at Early Time-Points

**Figure 5:**
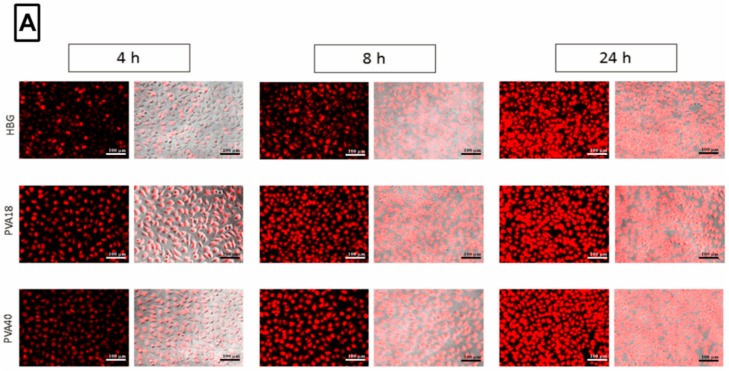

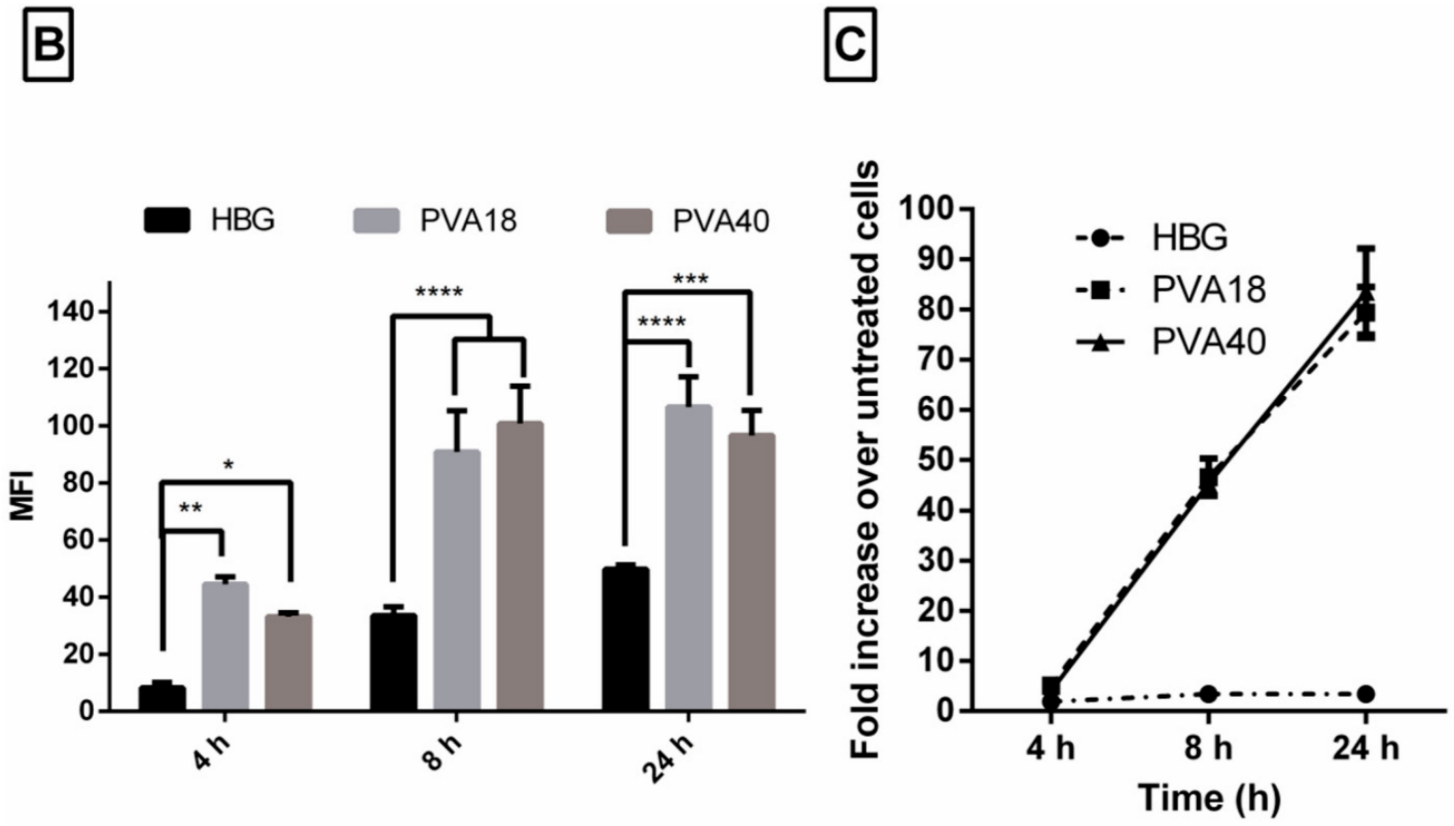
Effect of excipients on oligonucleotide (ON) uptake and luciferase activity levels at different time-points. (**A**) Uptake behavior after serum-associated transfection of HeLa Luc/705 cells using G2-RR/Lipofectin/Cy5 labeled ON complexes formulated in the HBG only or with excipient (PVA18 and PVA 40). (**B**,**C**) Mean fluorescence intensity (MFI) measured by flow cytometry, and Luciferase activity levels, respectively, after transfection with G2-RR PDLO-complexes formulated in HBG only or with excipient (PVA18 and PVA 40). Before imaging with fluorescence microscope, the live cells were rinsed with DMEM^®^ media without phenol red. The images shown represent both Cy5 channel and after merging with the phase contrast channels (magnification 20×, Scale bar = 100 μm). For MFI and luciferase activity results, Error bars = SEM (*n* ≥ 3), * *p* ≤ 0.05, ** *p* ≤ 0.01, *** *p* ≤ 0.001, and **** *p* ≤ 0.0001 (Two-way ANOVA, post hoc Fisher’s LSD).

We were interested to investigate if the excipients had any effect on the uptake of vector/ON complexes. Fluorescence microscopy images were taken for the cells at different time points after treatment with complexes containing labeled ON, with or without excipients. Although images show differences in uptake behavior at early time points (Figure 5A), measurements of the mean fluorescence intensity (MFI) upon merging, did not show any significant differences (data not shown). Hence, we utilized flow cytometry (FACS) for a more sensitive quantification for the uptake. FACS results (Figure 5B, Supplementary Figure S5) implied that the uptake behavior of the formulations was positively affected by the presence of the excipients. These findings were in accordance with that of Vasir and Labhasetwar [42] who suggested that association of low amounts of PVA on the surface of nanoparticles greatly enhanced their uptake and effect. Moreover, as seen from Figure 5B, the enhancement effect of the excipients on uptake was higher at early time points (4 h) compared to the later ones (24 h). For example, the PVA18-containing formulations displayed a 5-fold higher uptake values at 4 h, compared to the excipient-free formulations. However, at 24 h, the difference dropped to be only 2-fold. This might explain the more prevalent differences seen in the uptake behavior in fluorescence microscopy images at early time-points.

Additionally, luciferase activity was investigated at the same time-points used in the fluorescence microscopy and the FACS experiment. Luciferase levels were almost the same at 4 h in all formulations (Figure 5C). At later time-points, excipient-free formulations displayed almost the same luciferase values (~5-fold increase). However, formulations with excipients showed a greater increase in luciferase values, at 8 and 24 h (~45-, and 80-fold over background, respectively). This suggests that the excipients enhanced the efficacy of the formulations despite the surrounding intracellular barriers. PVA associated with nanoparticles has been reported to influence the interfacial properties of particles and hence their uptake, intracellular distribution, and expression of the delivered material [43]. It is possible that excipients either enhance the uptake of the particles and modulate their endosomal escape, or improve the stability of formulations against the degrading enzymes.

According to Wiśniewska et al. [44], the adsorption behavior of PVA to the surface of particles is governed by hydrogen bonding. Shifting pH from neutral to acidic would lead to protonation of the amino groups in the dendrimers and increase their hydrogen bonding with hydroxyl groups in PVA, hence enhancing adsorption.

In the light of the aforementioned study, we assumed that during the uptake process when pH was almost 7, the PVA molecules would be loosely adsorbed on the surface, sufficient to make the particle dispersed and enhance the uptake. However, the main protective feature of PVA would appear at the endosomal stage, as the surrounding acidic pH would cause protonation of N-termini of the peptide dendrimers (pKa around 6.5), leading to the formation of a more compactly adsorbed PVA layer that would enhance particle protection and maybe affect endosomal escape. This might explain the great difference in the luciferase values at different time-points in the presence and absence of PVA despite the small differences in uptake after 24 h (Figure 5).

### 3.7. Lead Excipients Enhance Efficacy of G2-RR PDLO-Complexes to Comparable Levels to L2000 in Different Reporter Cell Lines

**Figure 6:**
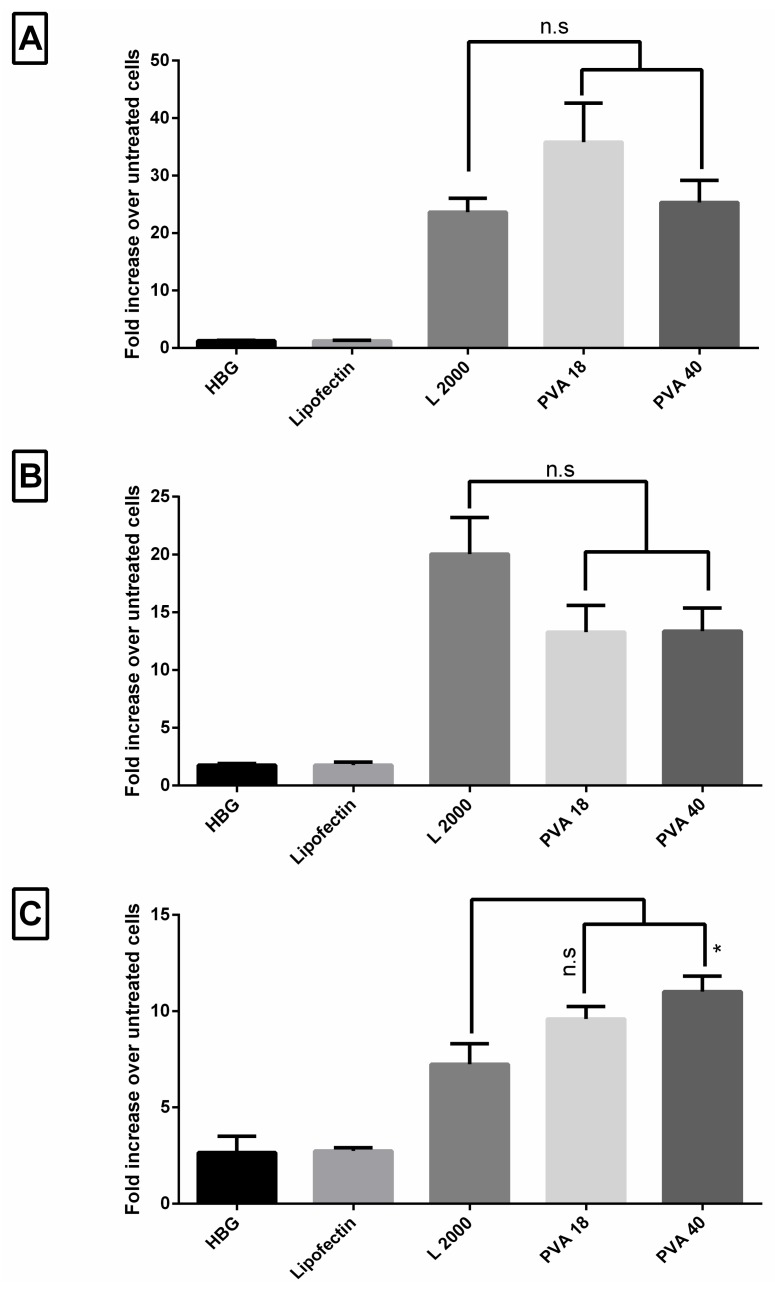
Effect of the selected excipients on transfection efficiency under serum conditions of G2-RR Dendrimer/Lipofectin/Oligonucleotide (PDLO) complexes in other reporter cell lines. Fold increase in the luciferase signal compared to the untreated in (**A**) HuH7_705, (**B**) Neuro 2a_705, and (**C**) U-2 OS_705 cells. Error bars = SEM (*n* ≥ 3), n.s.: non-significant, and * *p* ≤ 0.05 (one-way ANOVA, post hoc Fisher’s LSD against L2000).

Based on the investigation using the HeLa Luc/705 reporter cell lines, we studied the effect of adding of PVA excipients to the G2-RR PDLO-complexes, in the other reporter cell lines representing different tissues—U-2 OS_705, HuH7_705, and Neuro 2a_705. Transfection efficiencies under serum-associated conditions (HBG) are presented in Figure 6. In all tested cell lines, PVA enhanced the activity of formulations to be comparable to L2000. The only exception was in the U-2 OS_705 cells, as the PVA 40 addition produced a significantly higher activity (1.5-fold) as compared to L2000. This highlights the significance of cell-dependent behavior, upon delivery of splice-switching ONs with delivery vectors, which was previously reported by us and in other studies as well [17,45].

We have also tested the capability of the best excipients to enhance the activity of our formulations with splice-correcting ON for an additional human disease gene, *BTK*. We demonstrated that a mutation causing a pseudo-exon inclusion resulting in X-linked agammaglobulinemia, can be reverted at the pre-mRNA level, through splice-correction (Supplementary Figure S6). In addition, we tested formulations of 705 ON as an irrelevant oligonucleotide for the *BTK* system, to confirm that the effects observed is not due to an increased activity of the transcriptional machinery (Supplementary Figure S6).

### 3.8. Excipients Result in Smaller Sizes and Narrow Size Distribution of the Formulated Complexes

**Figure 7:**
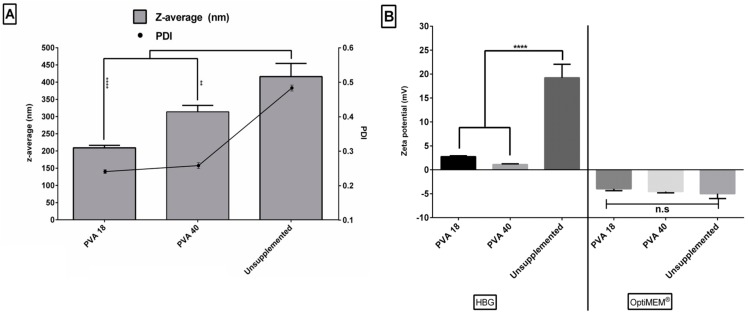
Evaluation of the excipient effect on the size and charge of the formulated complexes. (**A**) Mean Z-average diameter (Left Y axis) versus the polydispersity index (PDI) (Right Y axis) of different formulations, and (**B**) the zeta potential of selected PDLO-complexes formulated in HBG or OptiMEM^®^. Error bars = SEM (*n* ≥ 3), n.s.: non-significant, ** *p* ≤ 0.01, and **** *p* ≤ 0.0001 (one-way ANOVA, post hoc Fisher’s LSD against “unsupplemented”).

Figure 7A shows the sizes and polydispersity index values (PDI) of selected PDLO-complexes. Generally, excipient-free formulations displayed a larger average size (~400 nm) and PDI (~0.5) than those with excipients. This might be attributed to the higher incidence of aggregation in the formulations, due to the absence of stabilizing excipients. With regards to PVA excipients, PVA40 addition to formulations produced relatively larger sizes (300 nm) than PVA 18 (200 nm). This might be due to the larger molecular weight of the adsorbed PVA40 as compared to the PVA18. However, the PDI was decreased to 0.3, regardless of the type of the PVA, indicating a narrow size distribution and more homogeneity of the formulations. This would support the assumption of a formation of lower numbers of aggregates in the case of PVA. Finally, the zeta potential in the HBG buffer (Figure 7B) was lower in the formulations with added excipients, which might be explained by the polymer adsorption and displacement of the counter-ions in the Stern layer of the particles [46]. Figure 7B also depicts a shift in charge from positive to negative, upon changing the dilution vehicle from HBG to OptiMEM^®^. The effect of vehicle on zeta potential was previously reported on many occasions [17,47]. OptiMEM^®^ medium contains HEPES and other buffering salts that might have more influence on the zeta potential of particles, in contrast to HBG, in which the buffering salts were replaced with the less interacting glucose [48,49].

### 3.9. Lead Excipients Affect ON Biodistribution Profile in Mice

**Figure 8:**
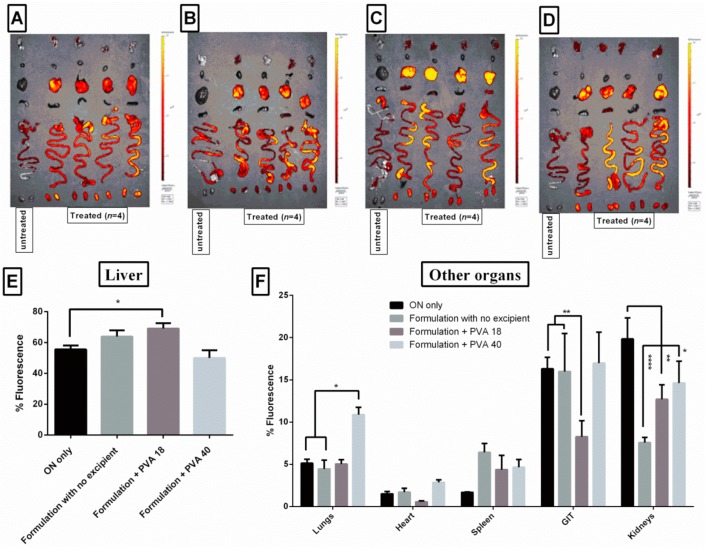
Biodistribution profile of G2-RR PDLO-complexes, with or without excipients. (**A**–**D**) Representative IVIS images of isolated organs, 24 h after intravenous injection of ON only, Formulation with no excipient, Formulation + PVA18, Formulation + PVA40, respectively. Organ arrangement from top to bottom—lungs, heart, liver, spleen, GIT, and kidneys. (**E**,**F**) Percentages of fluorescence signals in the liver and other organs, respectively (absolute fluorescence signals are represented in Supplementary Figure S7A). The final dose injected intravenously was 25-µg Alexa-568-labeled oligonucleotide/animal. The harvested organs were imaged (excitation 570 nm, emission 620 nm) using IVIS Spectrum (Perkin Elmer), 24 h after injections. In panels (**E**) and (**F**), Error bars = SEM (*n* = 4), * *p* ≤ 0.05, ** *p* ≤ 0.01, and **** *p* ≤ 0.0001 (one or two-way ANOVA, post hoc Fisher’s LSD).

Fluorescence imaging is commonly used to evaluate the biodistribution of nanoparticles formulations. It has the advantages of being a highly sensitive, non-invasive and relatively simple method to conduct in most of the cases [50]. ON from all injections showed the highest accumulation in liver, followed by the gastrointestinal tract (GIT) and kidneys (Figure 8). Although fluorescence signals in liver showed a greater accumulation in the presence of PVA 18 excipients (Figure 8A–D, Supplementary Figure S7A), PVA18-containing formulations displayed only a 15% higher relative fluorescent values in liver, as compared to free ON (Figure 8E). Contrarily, relative fluorescence values from the GIT and kidney were significantly decreased by 50%, in the case of the PVA18-containing formulations, as compared to free ON (Figure 8F). PVP 10 containing formulations displayed a similar pattern in liver and kidney accumulation (Supplementary Figure S7B–D). PVA40 containing formulations also showed less relative fluorescence values in kidney, but interestingly, they displayed two times relative fluorescence in lungs, compared to free ON (Figure 8F). We assumed that even though the enhancement in organ accumulation upon addition of excipients is moderate, it did not necessarily mean that they did not potentiate the activity. Addition of excipients showed a minimal impact on uptake, compared to the enhancement of activity in cell models (Figure 5). However, we were curious to investigate if there was an alteration in the biodistribution profile, as we previously reported for a different excipient [17].

Overall, the tested formulations were well tolerated in mice and there were no signs of toxicity. The results also revealed a desirable outcome of using the formulations as they decreased accumulation of phosphorothioate-ONs in kidneys and hence minimized toxicity. This also highlighted the superiority of the peptide over polymeric dendrimers to deliver phosphorothioate-ONs, as the latter were reported to accumulate in kidneys [51]. In addition, these results demonstrated the potential role of different excipients, whether they re-route from kidney to liver, or enhance the ON accumulation in other organs, such as lungs. Aforementioned biodistribution patterns might be more evident upon the use of higher doses, a limitation we faced in our study due to technical considerations and restrictions in the concentrations and volumes injected.

## 4. Conclusions

This study highlighted the influence of common excipients on uptake and transfection efficacy of formulated ON, under serum conditions. In our study, the performance of peptide dendrimer/lipid vector mixture was greatly enhanced by these excipients, without any toxic side effects. Formulations were successfully and efficiently able to deliver splice-switching ONs in the presence of serum, to different reporter cell lines. The enhancement was evaluated on both RNA and protein levels and emphasized the superior effects of polymers, compared to sugars on transfection efficiency. The excipients might have possibly enhanced the stability of particles and decreased their aggregation, or improved the protection and release of particles from the endosomal compartments. Finally, in vivo biodistribution showed the capability of these excipient-containing formulations to modestly change the pattern of ON biodistribution or target other organs using low doses, while the effect of higher doses remains to be explored. Although a comprehensive investigation of the mechanism of the added excipients was difficult due to the complexity of the system used (Dendrimer/lipid/ON), we tried to investigate the effect of excipients on the formulations on three levels—“physical”, where we measured the effect of the excipients on size and zeta potential; ‘cellular uptake’, investigated using florescence microscopy and flow-cytometry; and ‘activity’, investigated by luciferase assay and analysis of correction on the RNA level. To our understanding, it seemed that the excipient had more impact on the formulation’s activity than on their uptake process. Future studies will consider optimization of the system in addition to more investigations of the mechanism of these excipients, and their evaluation with different vectors or cargos.

## Figures and Tables

**Table 1:** List of excipients used in the study and composition.

Sugar Excipients	Final Concentration (*w/v*) *
Sucrose	9.25%
Glucose	5%
Lactose	9.75%
Arabinose	10%
Glucosamine	5%
Galactosamine	5%
*N*-acetyl Galactosamine	5%
Galactose	5%
Fructose	5%
(2-Hydroxypropyl)-β-cyclodextrin	10% HP-β-CD + 6.5% sucrose
**Polymer Excipients**	**Composition and Final Concentration (*w/v*)** *
PVP 10	5% PVP 10 + 6.3% sucrose
PVP 40	5% PVP 40 + 6.3% sucrose
PVP 55	5% PVP 55 + 6.3% sucrose
PVA 18	2% PVA 18 + 6.5% sucrose
PVA 40	2% PVA 40 + 6.5% sucrose
Tw 80	0.02% Tw80 + 9.25% sucrose
Tw 20	0.02% Tw20 + 9.25% sucrose
PF 68	10% PF68
PF 127	10% PF127

* The concentrations were selected to achieve isotonic solutions based on several references [22,23,24,25].

## Supplementary Materials

The following are available online at https://www.mdpi.com/1999-4923/11/12/666/s1, Figure S1: Excipients display no effect on transfection of oligonucleotide (ON) in the absence of transfection vectors. Figure S2: Excipients enhance transfection of oligonucleotide (ON) in PDLO-complexes through the lipid component. Figure S3: Evaluation of lyoprotective effect of the excipients. Figure S4: Treatment with formulations showed no effect on cell morphology or confluency. Figure S5: Flow cytometric quantification of Cy5-labeled ON uptake upon using different excipients. Figure S6. Lead excipients enhance activity of formulations with oligonucleotide (ON) targeting *BTK* gene U2OS/EGFPLucBTKint4mut reporter cell line. Figure S7: Biodistribution profile of G2-RR PDLO-complexes with different excipients.
