# Supplementary material for: Sugar and Polymer Excipients Enhance Uptake and Splice-Switching Activity of Peptide-Dendrimer/Lipid/Oligonucleotide Formulations
Source: Pharmaceutics. 2019 Dec 9;11(12):666. doi: 10.3390/pharmaceutics11120666 (PMC6955847; doi:10.3390/pharmaceutics11120666)
Supplement: Supplementary file 1 [file pharmaceutics-11-00666-s001.pdf]

pharmaceutics

Supplementary Materials: Sugar and Polymer Excipients Enhance Uptake and Splice-Switching Activity of Peptide-Dendrimer/Lipid/Oligonucleotide Formulations

Osama Saher, Taavi Lehto, Olof Gissberg, Dhanu Gupta, Oskar Gustafsson, Samir EL Andaloussi, Tamis Darbre, Karin E. Lundin, C. I. Edvard Smith and Rula Zain

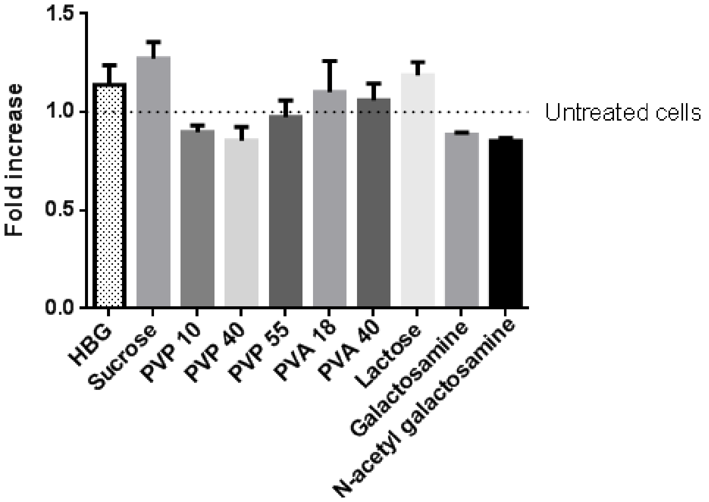

Figure S1. Excipients display no effect on transfection of oligonucleotide (ON) in the absence of transfection vectors**.** Fold increase in luciferase activity relative to the untreated cells using ONs formulated with selected excipients in absence of Lipofectin/dendrimer mixture. Evaluation was done under serum conditions. HeLa Luc/705 cells were seeded one day prior to transfection. Excipients and ON only, were mixed using HBG as the formulation vehicle. ON final concentration in the cultures was 0.1 μM and luciferase activity was analyzed 24 h after transfection and related to non-transfected cells. Each bar represents the mean with the standard error of the mean (SEM) of at least three independent experiments performed in triplicate ($n \geq 3$).

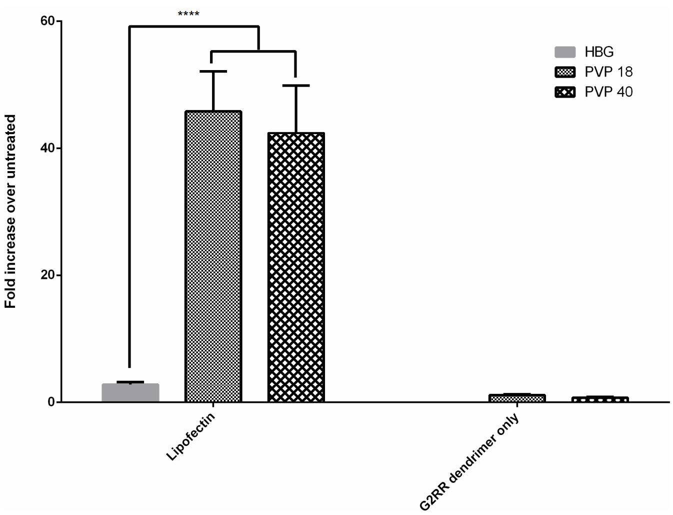

Figure S2. Excipients enhance transfection of oligonucleotide (ON) in PDLO-complexes through the lipid component Fold increase in luciferase activity relative to the untreated cells upon using selected excipients with either. Lipofectin/ON, or dendrimer/ON formulations. Evaluation was done under serum conditions. HeLa Luc/705 cells were seeded one day prior to transfection. Formulae (with or without excipients) were prepared in HBG buffer. ON final concentration was 0.1 μM and luciferase activity was analyzed 24 h after transfection and related to non-transfected cells. Each bar represents the mean and the standard error of the mean (SEM) of at least three independent experiments performed in triplicate (n ≥3). *P*-values were calculated using two-way ANOVA test and differences in fold increase were statistically compared using *post hoc* Fisher's LSD test (*****P* ≤ 0.0001).

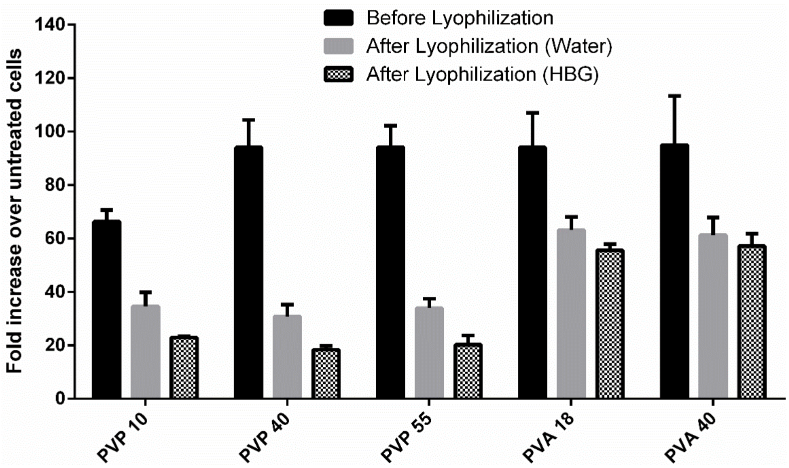

Figure S3. Evaluation of lyoprotective effect of the excipients. Luciferase activity levels (as fold increase over background) in HeLa Luc/705 cells after transfection with formulae together with selected excipients before and after their lyophilization. Lyophilization was conducted for 2 h using (MAXI-DRY LYO®) for formulations with or without excipients The dried products were reconstituted, added to cells, and luciferase measurements were performed 24 h after transfection with the reconstituted products. Each bar represents the mean with the standard error of the mean (SEM) of at least three independent experiments performed in triplicate ($n \geq 3$).

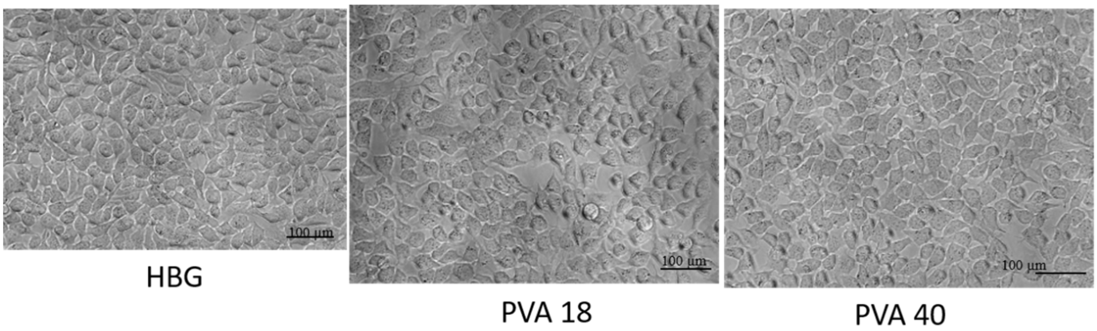

Figure S4. Treatment with formulations showed no effect on cell morphology or confluency. HeLa Luc/705 cells were seeded one day prior to transfection. Formulae (with or without excipients) were prepared in HBG buffer. ON final concentration was 0.1 µM. 24 h after transfection live cells were rinsed with DMEM® media without phenol red. Images were taken using a Phase-contrast microscope (magnification 20×, Scale bar = 100 µm).

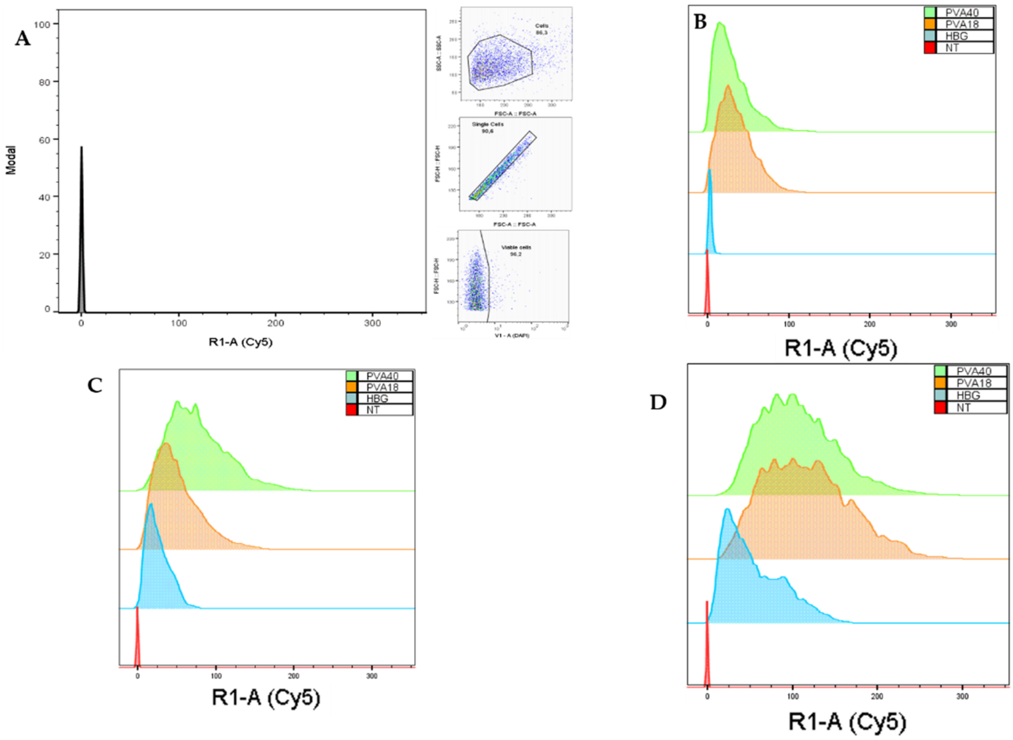

Figure S5. Flow cytometric quantification of Cy5-labelled ON uptake upon using different excipients. (A) Gating strategy for identification of single, viable cells and quantification of uptake of Cy5-labelled ON into untreated HeLa Luc/705 cells. (B,C,D) Histograms related to ON uptake from transfection complexes in the presence and absence of selected excipients after 4, 8 and 24 h, respectively. G2-RR PDLO-complexes with Cy5-labeled ONs formulated in HBG were tested with or without selected excipients. To measure uptake, cells were trypsinized, re-suspended in DMEM® media with 10% FBS (Invitrogen), and then stained with DAPI (0.1 µg/mL) before detection using MACSQuant Analyzer 10 instrument (Miltenyi Biotec, Bergisch Gladbach, Germany). DAPI was detected in channel V1 (450/50 nm) and Cy5 in channel R1 (655–730 nm). At least 2000 cells per well were acquired, and events were gated for single, viable cells. All data were analysed using FlowJo v10.5.3 (FlowJo, LLC) software.

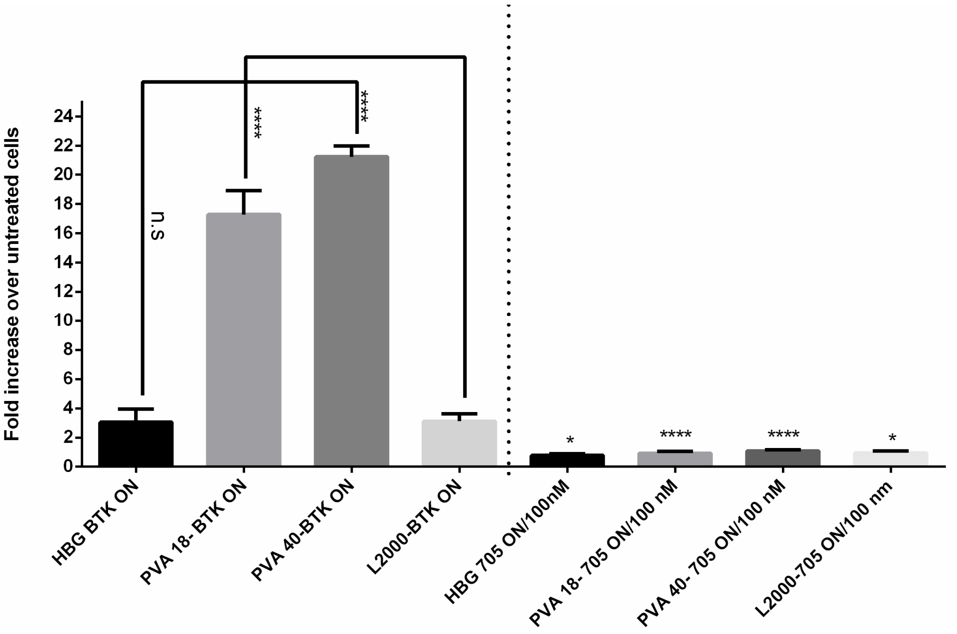

Figure S6. Lead excipients enhance activity of formulatons with oligonucleotide (ON) targeting the *BTK* gene as measured in the U2OS/EGFPLucBTKint4mut reporter cell line Fold increase in luciferase activity relative to the untreated cells upon using selected excipients with formulations containing ON targeting mutated *BTK* gene. Evaluation was done under serum conditions. U2OS/EGFPLucBTKint4mut reporter cells were seeded one day prior to transfection. Formulations (with or without excipients) were prepared in HBG buffer. ON final concentration was 25 nM and luciferase activity was analyzed 24 h after transfection and related to non-transfected cells. Each bar represents the mean and the standard error of the mean (SEM) of two independent experiments performed in duplicate (*n*=2). *P*-values were calculated using one-way ANOVA test and differences in fold increase were statistically compared using *post hoc* Fisher's LSD test against HBG BTK ON "left group" or against equivalent BTK ON formulation "right group" (*i.e.,* HBG 705 ON/100 nM against HBG BTK ON and, PVA 18-705 ON/100 nM against PVA 18-BTK ON, *etc.*) (n.s: non-significant, *$P \leq$ 0.05, **** $P \leq 0.0001$).

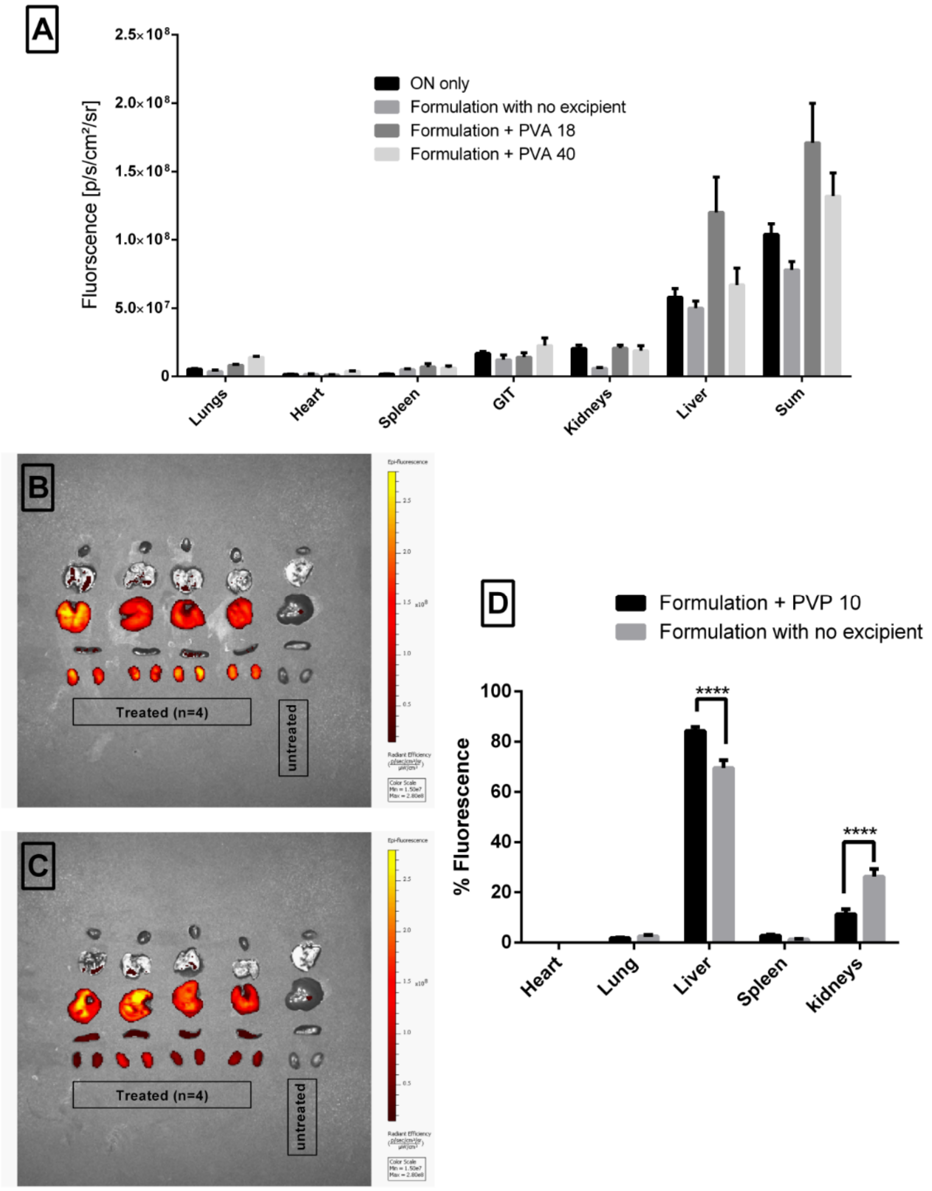

Figure S7. Biodistribution profile of G2-RR PDLO-complexes with different excipients. (**A**) Absolute fluorescent signals of the results mentioned in Figure 8 E& F. (**B** and **C**) Representative IVIS images of isolated organs (from top to bottom: Heart, lungs, liver, spleen, and kidneys) 24 h after intravenous injection of ON only, Formulation + PVP 10, respectively. (**D**) Percentages of fluorescent signals in the isolated organs represented in images (**B** and **C**). In all experiments, injection dose was 25 µg Alexa-568 labeled oligonucleotide/animal formulated as a PDLO-complex employing G2RR alone or with different excipients. ON mixed with HBG buffer is also included as positive control. The animals were sacrificed and harvested organs were imaged the next day (excitation 570 nm, emission 620 nm) using IVIS Spectrum (Perkin Elmer). Values represent the mean with the standard error of the mean (SEM). *P*-values were calculated by two-way ANOVA test and differences were compared using *post hoc* Fisher's LSD test. (**** $P \leq 0.0001$).
